# Assessing the agreement between the partners at care transitions measure and the care transitions measure for elderly patients with chronic diseases

**DOI:** 10.1186/s12913-023-09228-7

**Published:** 2023-05-09

**Authors:** La-mei Liu, Meng-jie Sun, Meng-ting Liu, Jia-nan Wang, Yi-zhen Zhang, Ronnell D Dela Rosa

**Affiliations:** 1 School of Nursing and Health, Zhengzhou University, 100 Science Avenue, High-Tech District, Zhengzhou City, 450000 Henan Province China; 2 Shanxi Bethune Hospital, 99 Longcheng Street, Xiaodian District, Taiyuan City, 030032 Shanxi Province China; 3 School of Nursing, Philippine Women’s University, Taft Avenue, Malate, 1004 Manila, Philippines; 4 College of Nursing and Midwifery, Bataan Peninsula State University, City of Balanga 2100, Bataan, Philippines

**Keywords:** Elderly, Chronic diseases, Transitional care, Quality of care, Instrument

## Abstract

**Background:**

Elderly patients with chronic diseases are very vulnerable during the transition from hospital to home and have a high need for transitional care. The quality of transitional care is closely related to patient health outcomes. Using appropriate scales to evaluate the quality of transitional care is important for efforts aimed at improving it. The study aimed to analyze the consistency between the Chinese version of the Partners at Care Transitions Measure (PACT-M) and the Care Transition Measure (CTM) in assessing the quality of transition care in elderly patients with chronic diseases.

**Methods:**

This is a cross-sectional study, we used a convenience sampling method to investigate patients with chronic diseases aged ≧ 65 years who were about to be discharged from the three affiliated hospitals of Zhengzhou University in Henan Province, from August 2021 to May 2022. The sample consisted of 196 elderly patients with chronic diseases. Data were collected using a demographic survey, PACT-M, and CTM. We used EpiData 3.1 software for systematic logical error checking, SPSS 21.0 to analyze the data, and the Bland–Altman analysis to analyze the consistency of the two scales.

**Results:**

The mean total scores for PACT-M and CTM were 65.52 ± 6.23 and 52.07 ± 7.26, respectively. The 95% confidence interval (*CI*) for the mean difference and ratios were (-31.52, 4.61) and (0.85, 1.72), with 3.57% and 5.10% of the points outside the 95% *CI* limits, separately.

**Conclusions:**

The difference analysis of Bland–Altman showed a good consistency of the two scales, while the rate analysis did not meet the a priori definition of good consistency, but it is very close to 5%. Therefore, the consistency of the two scales in assessing the quality of transitional care for elderly patients with chronic diseases needs to be further validated.

## Background

The aging of the population is considered a global social and health challenge [1]. The aging situation is even more serious in China, the results of the seventh population census show that by the end of 2020, the population of China aged 60 and over was about 264 million, and the population aged 65 years and older was about 191 million, accounting for 18.7% and 13.5% of the total population respectively [2]. In an aging population, the incidence of chronic diseases in the elderly is also increasing [3]. Almost 180 million older people in China suffer from chronic diseases, and multiple chronic diseases (suffering from two or more chronic diseases) have gradually become the main group of chronic disease patients [4]. Chronic diseases in China are characterized by “a large number of patients, high medical costs, long illness times, and large service demands”, which require a lot of investment in care costs and medical insurance costs, and are becoming the biggest public health problem that afflicts Chinese society and its families [5].

To meet the medical and healthcare needs of elderly patients with chronic diseases and reduce the social and economic burden caused by chronic diseases, the Chinese government has issued a series of policies. For example, make full use of existing health resources, shorten the average hospital stay, speed up hospital bed turnover, and improve the operational efficiency of medical institutions [6]. Strengthen the rehabilitation and nursing service capabilities of primary medical and health institutions, provide services such as elderly care, family beds, and home-based elderly care, and promote the extension of medical and health services to communities and families [3, 7]. Under the guidance of the above-mentioned policies, the total amount of medical and health service resources in China has continued to grow and the level of medical technology and medical quality has been continuously improved. Currently, a health and medical service network has been formed that covers both urban and rural areas [8]. Most elderly patients with chronic diseases can receive transitional care after returning home from the hospital.

With the development of transitional care, more tools have been developed to assess the quality of transitional care. These tools include the Care Transitions Measure (CTM) [9, 10], the Questionnaire to Measure Older People’s Experience of the Transition Care Program(QMOPETCP) [11], the Partners at Care Transitions Measure (PACT-M) [12, 13], the Discharge Care Experiences Survey (DICARES) [14], the Patient Continuity of Care Questionnaire (PCCQ) [15] and the Nijmegen Continuity Questionnaire (NCQ) [16]. All of above the scales can assess the quality of transitional care from the patient's perspective, and their reliability and validity have been validated. However, there are some differences, such as CTM, PACT-M, and DICARE for assessing the quality of transitional care from hospital to home, and QMOPETCP for assessing the quality of transitional care from hospital to non-acute care settings (e.g., nursing homes, care homes, etc.). And PCCQ and NCQ for assessing the experience and satisfaction with continuity of care. Since most elderly patients with chronic diseases will return home directly after discharge from the hospital in China, and only the CTM and PACT-M have Chinese versions, these two tools are the most commonly used in China [8, 17]. In addition, since the two scales are very similar, they are often used interchangeably to assess the quality of transitional care. However, whether the two scales can be used interchangeably and whether their results are consistent have not been reported. Therefore, the purpose of this study was to test the agreement between PACT-M and CTM and to further analyze the differences between the two scales to help researchers or medical personnel select the most appropriate scales according to the purpose of study or application.

## Methods

### Design and setting

This is a cross-sectional study design. We used the PACT-M and CTM to evaluate the quality of transition care in three affiliated hospitals of Zhengzhou University in Henan Province. The three hospitals we surveyed are all first-class and tertiary general hospitals with departments such as cardiovascular, respiratory, endocrinology, neurology, oncology, and urology. These hospitals serve enough elderly patients to provide a sufficient sample for recruitment.

### Data collection

We administered the PACT-M_1_ face-to-face with patients when they were about to be discharged from the hospital, and the PACT-M_2_ and CTM questionnaire by telephone within 1 month after discharge. A total of 196 patients participated in the survey. The inclusion criteria for the patients were as follows: (1) the patients were chronic patients aged 65 years or older; (2) the patients could be contacted by telephone after discharge; (3) the patients were discharged to home rather than long-term care facilities; (4) the patients had no obvious cognitive or language disabilities; (5) the patients were willing to participate in the survey.

Data collection was carried out by 3 post-graduate students with uniform training. After obtaining the consent of the relevant hospital departments, department heads, and patients, patients were first informed of the purpose and importance of this study, and each patient was required to sign an informed consent form. After that, we used PACT-M_1_ to investigate elderly patients with chronic diseases who met the inclusion criteria to understand the patient’s experiences of care transition in the immediate post-discharge period. To protect patient privacy, patients completed the questionnaire anonymously but were required to provide their contact information for follow-up. The researcher used a unified instructional language to explain the purpose of the survey, and the methods and precautions for filling out the questionnaire to the respondents. One month after using the PACT-M_1_ survey, patients who participated in the first survey were followed by telephone using the PACT-M_2_ to evaluate their experience of managing health and care at home and the CTM to assess the experience of transition care of patients. The survey was conducted between August 1, 2021, and May 30, 2022.

Regarding the handling of missing data, in the first phase of the survey using PACT-M_1_, the survey was conducted face-to-face. After each patient completed the questionnaire, the investigator performed a preliminary check to ensure that the questionnaire was complete. If there were any missing pieces, the investigator communicated with the patient to determine why they did not answer the question. If the missing answer was because the patient had missed the questions, the investigator asked the patient to complete the information. If the patient did not know how to answer, the investigator explained the meaning of the question to the patient and then asked the patient to complete the questionnaire. Therefore, there were no missing data in the first phase of the survey. In the second phase of the survey using PACT-M_2_ and CTM-15, the survey was conducted by telephone and both PACT-M_2_ and CTM-15 were filled in by investigators who asked patients and filled in according to the actual situation of the patients. Therefore, there are no missing values in the survey for both phases.

### Instruments

#### The demographic survey

It is designed by the researchers and mainly includes two parts: (1) Demographic information: gender, age, marital status, education level, monthly household income, occupation before retirement, type of medical insurance, living status, etc.; (2) Disease-related medical information: chronic disease status, disease progression, whether there is someone to take care of them, etc.

#### The Chinese version of PACT-M

The Chinese version of the PACT-M is developed from the original version of the PACT-M, which is a patient-reported questionnaire for evaluation of the quality and safety of care transitions from hospital to home [12, 13]. It consists of two parts, PACT-M_1_ and PACT-M_2_. The PACT-M_1_ includes 9 items in two dimensions and is used to capture the patient’s experience in the immediate post-discharge period. The PACT-M_2_ includes 8 items in two dimensions and is used to assess long-term experience in managing health and care at home. Each item was rated on a 5-point Likert scale from 1 to 5 (“strongly disagree” to “strongly agree”), the score range of PACT-M_1_ is 0 ~ 45 points, and the score range of PACT-M2 is 0 ~ 40 points. The total PACT-M score is the sum of the two parts. A higher score for the total PACT-M indicates better quality of care during the transition. There is also a “not applicable” option but was not included in the total score. The Cronbach’s alpha values for PACT-M_1_ and PACT-M_2_ were 0.802 and 0.741, and the test–retest reliability values were 0.885 and 0.837. The results of both the exploratory factor analysis (EFA) and the confirmatory factor analysis (CFA) showed that both parts of the PACT contained two dimensions, the item content validity index and scale content validity index values of PACT-M_1_ and PACT-M_2_ were 1.0 [8].

#### The Chinese version of CTM

The Chinese version of CTM is developed from the original version of the CTM, which is a self-report measure of the quality of care transitions that capture the patient's perspective [9]. It consists of 17 items in four dimensions (self-care preparation, written plan, doctor-nurse-patient communication, and symptom management). Each item is assessed on a 4-point scale ranging from 1 “strongly disagree” to 4 “strongly agree”, with higher scores indicating better quality of care transitions. The Cronbach’s α for the total scale was 0.85, and the Cronbach’s α for each factor ranged from 0.61 and 0.89, the content validity index for the total scale was 0.99, and the CVI for each item of the scale ranged from 0.80 to 1.00. The Chinese version of CTM is a reliable and valid measure of evaluating the quality of care transition among patients in Mainland China [17].

### Statistical analysis

**Fig. 1:**
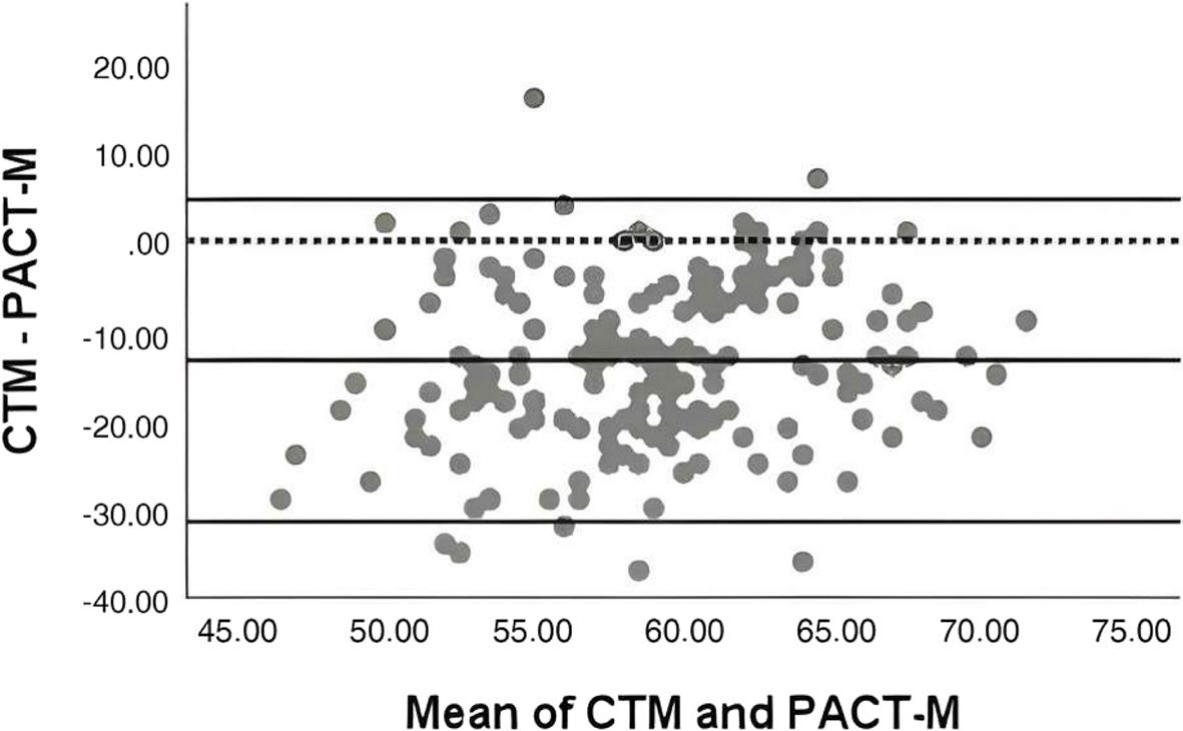
Bland–Altman analysis plot of the difference between the PACT-M and CTM. The four-parameter lines from top to bottom are the maximum value of 95%*CI*, the mean of the difference, the theoretical line (0), and the minimum value of 95%*CI*

**Fig. 2:**
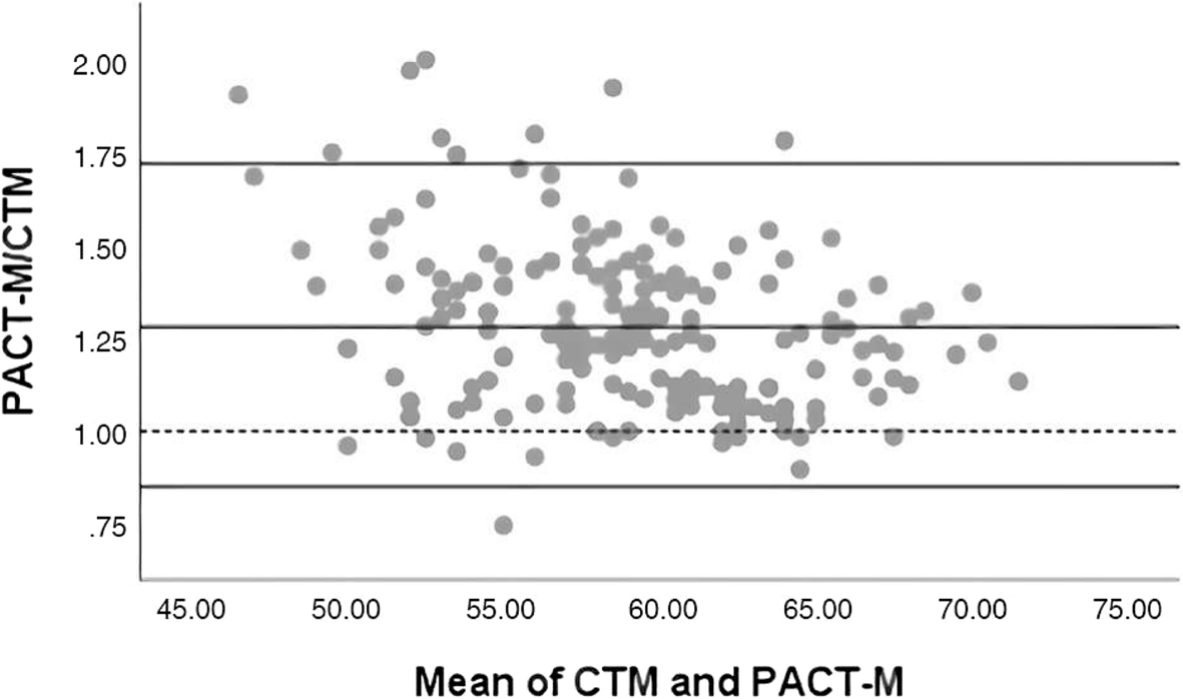
Bland–Altman analysis plot of the ratio between the PACT-M and CTM. The four-parameter lines from top to bottom are the maximum value of the 95% *CI*, the mean of the ratio, the theoretical line (1), and the minimum value of 95% *CI*

The returned questionnaires were uniformly numbered, the data was entered by two people, and EpiData 3.1 software was used for systematic logical error checking. Data were statistically analyzed using SPSS 21.0. The characteristics of participants were described using frequencies and percentages, and PACT-M and CTM-15 scores were described using means and standard deviations. Currently, there are no uniform criteria to assess the quality of transition care in elderly patients with chronic diseases, and it was not possible to compare the CTM and PACT-M scales with the criteria to verify their consistency. The bland–Altman analysis is a method to quantify the agreement between two quantitative measurements by constructing limits of agreement, and the results of the analysis can be visualized in a Bland–Altman plot [18]. The Bland–Altman plot is a two-dimensional right-angle coordinate (see Figs. 1 and 2). The horizontal axis x and the vertical axis y represent the mean and the difference (or ratio) of the two measurement methods, respectively. The upper and lower horizontal solid lines represent the maximum and minimum values of the 95% *CI*, the middle solid line represents the mean of the difference (or ratio), and the dashed line represents the mean of the difference of 0. The higher the agreement between the two measures, the closer the solid line representing the mean of the difference (ratio) is to the dotted line representing the mean of the difference of 0. The consistency of the two methods evaluated was evaluated based on the maximum number of 95% *CI* maximum and minimum data points and the maximum difference within the limits of agreement, as well as the degree of clinical acceptability.

### Ethical Statement

The study was carried out following the Declaration of Helsinki Ethics Principles for Medical Research Involving Human Subjects and was approved by the Ethics committee of Zhengzhou University in China (approval number: ZZUIRB2021-78) before the study started. The study was approved by the hospitals’ managers, and consent was obtained from all participants. The patients were guaranteed confidentiality, anonymity, and the right to withdraw from the study at any time.

## Results

### Characteristics of the participants

In this study, a total of 210 questionnaires were distributed. 196 patients completed the survey (response rate of 93.3%). The age of the patients is 65 to 104 years. The majority of the participants were female (52.6%). The educational level was generally low, with 67.9% of the participants in junior high school and below. Most of the participants (87.2%) lived with their spouses or other family members. The top five diseases of the participants were heart disease, diabetes mellitus, hypertension, chronic lung disease, and cancer. Further demographic characteristics are presented in Table 1.

**Table 1:** Characteristics of the participants (*n* = 196)

**Variables**	*N* = 196	Percent (%)
**Gender**		
Female	103	52.6
Male	93	47.4
**Age**		
65 ~	119	60.7
75 ~	62	32.1
85 ~	11	5.6
95 ~	4	2.0
**Married**		
Yes	174	88.8
No	22	11.2
**Education level**		
Junior high school and below	133	67.9
High school and Junior college	51	26.0
Undergraduate	9	4.6
Graduate student and above	3	1.5
**Household income per month (Yuan)**		
< 1000	38	19.4
1000 ~	47	24.0
3000 ~	55	28.1
5000 ~	56	28.6
**Residence**		
City	99	50.5
Town	37	18.9
Rural	60	30.6
**Preretirement occupation**		
Worker	65	33.2
Farmer	73	37.2
Enterprise (business) unit	11	5.6
Individual household	33	16.8
Military	7	3.6
Medical and nursing personnel	6	3.1
No fixed work	1	0.5
**With or without a caregiver**		
With	171	87.2
Without	25	12.8
**If there is a caregiver, the caregiver is**		
Spouse	78	39.8
Children	82	41.8
Other	11	5.6
**Religious affiliation**		
Yes	24	12.2
No	172	87.8
**Ethnic group**		
Han nationality	175	89.3
Other	21	10.7
**Medical insurance**		
Yes	189	96.4
No	7	3.6
**Living situation**		
Living alone	13	6.6
With spouse only	63	32.1
Living with more than one person	120	61.2
**Disease type**		
Chronic lung disease	20	10.2
Cancer	11	5.6
Diabetes mellitus	26	13.3
Heart disease	37	18.9
Kidney disease	5	2.6
Stroke	7	3.6
Hypertension	24	12.2
Other diseases	66	33.7

#### The score of the Chinese version of PACT-M

The total PACT-M score, PACT-M_1_, PACT-M_2_, and the scores of each entry are shown in Table 2.

**Table 2:** Results of the scoring of patients with PACT-M

PACT-M	Item	Score $(\overline{{\varvec{x}}}\pm \mathbf{s})$
		**65.52 ± 6.23**
**PACT-M**_**1**_	**Patients’ experiences in the preparation for transition**	**33.82 ± 5.34**
	**Perceived health management support at the hospital**	**23.06 ± 3.65**
	1. I felt I could ask staff questions about what will happen after going home	4.08 ± 0.91
	2. Before leaving the hospital, I was confident I understood how to manage my medication	3.93 ± 0.90
	3. While I was in the hospital, there was someone who I could talk to if I was worried	3.81 ± 0.91
	4. Before leaving the hospital, I felt confident about what to do if my health became worse at home	3.81 ± 0.87
	5. I feel that my concerns regarding my health were addressed before I went home	3.68 ± 0.91
	6. I felt prepared to be at home	3.76 ± 0.99
	**Received information and support at the hospital**	**10.76 ± 2.38**
	1. While I was in the hospital, staff helped me to prepare for things that I might find difficult when I go back home (such as walking, cooking, showering, shopping, or being in pain)	3.82 ± 0.85
	2. Before leaving the hospital, I understood how to get help (or support) from my community services (e.g., doctors, nurses, and home care staff)	3.31 ± 1.21
	3. Before leaving the hospital, I knew what arrangements had been made to support me at home (for example, home care or community care visits)	3.63 ± 0.94
**PACT-M**_**2**_	**Patients’ experiences with managing their health and care at home**	**30.15 ± 4.94**
	**Perceived health management support at home**	**18.61 ± 3.34**
	1. I feel I have the support I need from community health services (e.g., doctors, nurses, and home care staff)	3.38 ± 1.19
	2. I feel confident about managing my health at home	3.72 ± 0.95
	3. I feel that there is someone I can talk to about my worries (for example, health care staff or my family)	3.81 ± 0.98
	4. I know what to do and who to contact if my health gets worse	3.85 ± 0.93
	5. I feel I can now manage my care safely at home	3.85 ± 0.89
	**Home health management**	**11.54 ± 2.01**
	1. I know who to contact if I have any questions about my health and healthcare	4.00 ± 0.95
	2. I know how to manage my medications	3.82 ± 1.01
	3. I have the necessary support to manage everyday activities (e.g., cooking, cleaning, buying food, showering, walking, and dressing)	3.72 ± 0.93

#### The score of the Chinese version of CTM

The total scores of CTM and the scores of the four dimensions are shown in Table 3.

**Table 3:** Results of the CTM scoring patients

The Chinese version of CTM	Score $(\overline{{\varvec{x}}}\pm \mathbf{s})$
	**52.07 ± 7.26**
**Self-care preparation**	**24.85 ± 3.82**
1. Acquire the required knowledge	2.97 ± 0.75
2. Know the disease condition	3.07 ± 0.69
3. Know the factors that can improve/worsen the condition	3.33 ± 0.55
4. Know the precautions for health management	3.26 ± 0.67
5. Have confidence in managing health	3.11 ± 0.73
6. Be confident that you can do what the medical staff tells you	3.07 ± 0.72
7. Know the purpose of the medication	2.87 ± 0.88
8. Know how to use the medicine	3.18 ± 0.70
**Written plan**	**5.96 ± 1.42**
1. Written self-care materials	2.91 ± 0.84
2. Written follow-up materials	3.06 ± 0.75
**Doctor-nurse-patient communication**	**12.56 ± 1.90**
1. Agree with the health goals set by the medical staff	3.18 ± 0.57
2. Consider the patient’s input when developing a care plan	3.13 ± 0.63
3. Consider patient opinions when recommending medical places	3.16 ± 0.66
4. Know how to contact medical staff	3.10 ± 0.71
**Symptom management**	**8.69 ± 1.76**
1. Know which signs and symptoms to monitor	3.11 ± 0.72
2. Know how to deal with uncomfortable symptoms	3.05 ± 0.67
3. Know the adverse effects of the drug	2.54 ± 0.93

### Bland–Altman analysis with the difference

We took the mean of the total scores for PACT-M and CTM as the abscissa and the difference as the ordinate. The mean difference is (-13.45 ± 9.22) points, the maximum value of the difference is 16, and the minimum value is -37. The 95% *CI* for the mean difference was (-31.52, 4.61). The four-parameter lines from top to bottom are the maximum value of 95% *CI*, the mean difference, the theoretical line (0), and the minimum value of 3.6% *CI*. 3.57% (7/196) of the points are outside the limits of 95% agreement, see Fig. 1(Bland–Altman analysis plot of the difference between the PACT-M and CTM).

### Bland–Altman analysis with ratios

We took the mean of the total scores of PACT-M and CTM as the abscissa and the ratio as the ordinate. The mean of the ratio is (1.28 ± 0.22) points, the maximum value of the ratio is 2.00, and the minimum value is 0.75. The 95% *CI* for the mean of the ratios was (0.85, 1.72) The four-parameter lines from top to bottom are the maximum value of the 95% *CI*, the mean of the ratio, the theoretical line (1), and the minimum value of the 95% *CI*. 5.10% (10/196) points are outside the bounds of 95% agreement, see Fig. 2 (Bland–Altman analysis plot of the ratio between the PACT-M and CTM).

## Discussion

In this study, Through Bland–Altman analysis of the difference, there are 7 points outside the 95% consistency limit, accounting for 3.57% of the total points. The mean of both PACT-M and CTM was 58.79 ± 4.95, the mean of the difference was -13.45 ± 9.22, the maximum value of the difference was 16, and the minimum value was -37. Through Bland–Altman analysis with ratios, there were 10 points outside the 95% consistency limit, accounting for 5.10% of the total points. The mean ratio was 1.28 ± 0.22, the maximum value of the ratio was 2.00, and the minimum value was 0.75, indicating that the measured value of PACT-M was 0.75 to 2.0 times higher than that of CTM. The total score range of the PACT-M scale is 17 ~ 85, and the total score of the CTM is 17 ~ 68. In the Bland–Altman ratio analysis, 10 points exceeded the 95% agreement limit, accounting for 5.10% of the total score, slightly exceeding the 5% limit. Combining the different range and ratio ranges within the 95% consistency limit, the points outside the 95% consistency limit, and the acceptability in practical applications, it is concluded that the different analyses of Bland–Altman showed a good consistency of the two scales, while the rate analysis did not meet the a priori definition of good consistency, but the ratio analysis is very close to 5%. Therefore, the consistency of the two scales in assessing the quality of transitional care for elderly patients with chronic diseases needs to be further validated.

The reason the rate analysis did not meet the a priori definition of good consistency may be related to the following reasons. First, it has been shown that Bland–Altman analysis results are sample size dependent, with the 95% *CI* becoming larger and the likelihood of the maximum allowable difference being within 95 *CI* increasing when the sample size is small. In contrast, the *CI* becomes smaller when the sample size is larger and the likelihood that the maximum allowable difference is within 95 *CI* decreases [19, 20]. However, the estimation of sample size for Bland–Altman analysis has not been reported so far. We used G-power analysis to estimate sample size in this study, which provides the effect size conventions as “small,” “medium” and “large”, based on Cohen’s suggestions, these provide conventional effect size values that are different for different tests [21]. We chose "medium" for the calculation in this study, considering the relationship between Bland–Altman analysis and sample size, we may choose "large" for the calculation of sample size in the future to further verify the consistency of the two scales. Second, it may be related to the characteristics of the two scales. Although both PACT-M and CTM assess the quality of care from the hospital to home from the patient’s perspective, they both have 17 items in four dimensions and some of the evaluation contents are similar, such as patient medication management, symptom management, communication with medical staff and seeking social support. However, these two scales also have their characteristics. For example, CTM is developed using qualitative research, it includes four dimensions but these four dimensions make up the overarching unidimensional construct evaluating the overall quality of transitional care and are summarized as one total score [10]. The PACT-M was developed based on literature analysis and qualitative research, it consists of PACT-M_1_ and PACT-M_2_. PACT-M_1_ to capture the immediate post-discharge period and PACT-M_2_ to assess the experience of managing care at home [13], this is different from the four dimensions of CTM as a total. From the perspective of evaluation content, CTM focuses on evaluating the patient’s mastery of self-management, medication management, symptom management, etc. during the transition from hospital to home, while PACT-M focuses on assessing the patient’s perceived or received transitional care, this is also different. From the assessment setting, CTM can be used to assess the patient’s transitional care experience from the hospital to a skilled nursing facility or other medical institutions, while PACT-M focuses primarily on patients from hospital to home, which is not suitable for other settings. The time points of the survey are also different. The CTM is evaluated within 2 to 6 weeks after discharge, while the two parts of PACT-M are evaluated at the time of discharge and within 1 month after discharge, respectively. Therefore, given the results of the Bland–Altman analysis and the characteristics of the two scales, their application should be selected according to the actual situation. For example, the PACT-M is only used to assess the quality of transitional care from hospital to home, while the CTM can also be used to evaluate patients' transition from hospital to healthcare facilities such as nursing homes and communities, which has a wider range of applications. In terms of evaluation content, CTM focuses on evaluating the specific content of patients' self-management, medication management, and symptom management during the transition period, while PACT-M focuses on evaluating patients' perceptions and experiences of transitional care during the transition period. In terms of evaluation timing, CTM is usually evaluated within 2–6 weeks after the patient's discharge, whereas PACT-M is evaluated at discharge and within 1 month after discharge, respectively. Therefore, researchers or medical personnel should choose the most appropriate scale according to the purpose of research or application and the respective characteristics of the scale.

Regarding the quality of transitional care, the total score for PACT-M was 47 ~ 82 points (65.52 ± 6.23), and the total score for CTM was 32 ~ 68 (52.07 ± 7.26). The CTM score was lower than the findings (63.1 ± 15.4) of Anu Birla Bakshi et al.'s [22] on 165 Chinese-speaking respondents in Hong Kong, and the PACT-M has not been reported to be applied for evaluating the quality of transitional care, except for its development and reliability and validity tests by the authors [12, 13]. The reasons for the low CTM scores in this study may be related to the earlier and more mature development of transitional care in Hong Kong. As early as the 1990s, Hong Kong has begun to explore the development of transitional care and has now created the 4-Cs based on the continuity (Continuity), comprehensiveness (Comprehensiveness), coordination (Coordination), and cooperation (Collaboration) characteristics of the 4-Cs model of transitional care [23]. In mainland China, transitional care has been incorporated into the national nursing development plan as one of the main tasks for the development of nursing in China from 2016–2020, but how to implement and promote it still needs further study [24]. Nevertheless, both the PACT-M and CTM scales measured the quality of transition care for elderly patients with chronic diseases at an upper-middle level. This may be related to a series of health policies issued by the state in recent years, such as promoting the construction of urban medical alliances [25], county medical alliances [26], and promoting orderly sinking of high-quality medical resources [27], so that more patients can obtain the corresponding high-quality nursing services after discharge. The National Health and Medical Commission held a press conference on 28 July 2022, proposing to meet the health needs of people in all aspects of the whole life cycle by building a high-quality and efficient integrated medical and health service system [28]. Under the guidance of the above policies, China’s medical and health service system that covers both urban and rural areas has been further improved, the fairness and accessibility of health services have improved significantly, and the quality and efficiency of services have continued to improve. Transitional care is a part of the medical and health service system, and major hospitals are also actively exploring and trying to adopt a variety of methods, such as the establishment of telemedicine apps, WeChat apps, and other platforms to provide patients with continuous care services, which to a large extent satisfies the Transitional care needs of patients, and ensure the improvement of the quality of transitional care.

### Strengths and limitations

There are several limitations to our study. First, the participants came from 3 tertiary A-level hospitals in Zhengzhou, China. The level of the hospital medical service is the best in Henan Province. They represent a better level of transitional care services, but the results are not representative of the level of quality transitional care in secondary hospitals or communities. Second, the study sample size was small and the inclusion criteria excluded patients with psychiatric impairment, cognitive impairment, or inability to communicate due to visual or hearing impairment. Therefore, the representativeness and applicability of the research results are limited, which may also be an important reason for the slightly higher 95% CI of the Bland–Altman analysis with ratios, which needs to be further verified by expanding the sample size. Finally, because the patients were elderly, to reduce missing data, About less than 10% of the patients asked questions about the scale questions, and the investigators interpreted the meaning of the questions according to the patients, which could lead to a potential bias. Based on these limitations, it is recommended that future studies should further expand the sample to verify the consistency of the two scales.

## Conclusions

The results of this study show that the Chinese version of PACT-M is a scale with good reliability and validity by comparing it with CTM, although the ratio analysis didn’t meet the prior definition of good consistency, the ratio analysis is very close to 5%, this may have something to do with the small sample size of this study. Therefore, the consistency of the two scales still needs to be verified by further increasing the sample size in future studies. It also suggests that although both scales can assess the quality of transitional care from the patient's perspective, there are still some differences in the content of the evaluation, the evaluation time, and the evaluation environment. Researchers or clinical staff should adopt the most appropriate scale for the survey when using these two scales.

## Data Availability

The data sets used and analyzed during the current study are available from the first author upon reasonable request.

## Abbreviations

PACT-M: Partners at Care Transitions Measure
CTM: Care Transitions Measure
